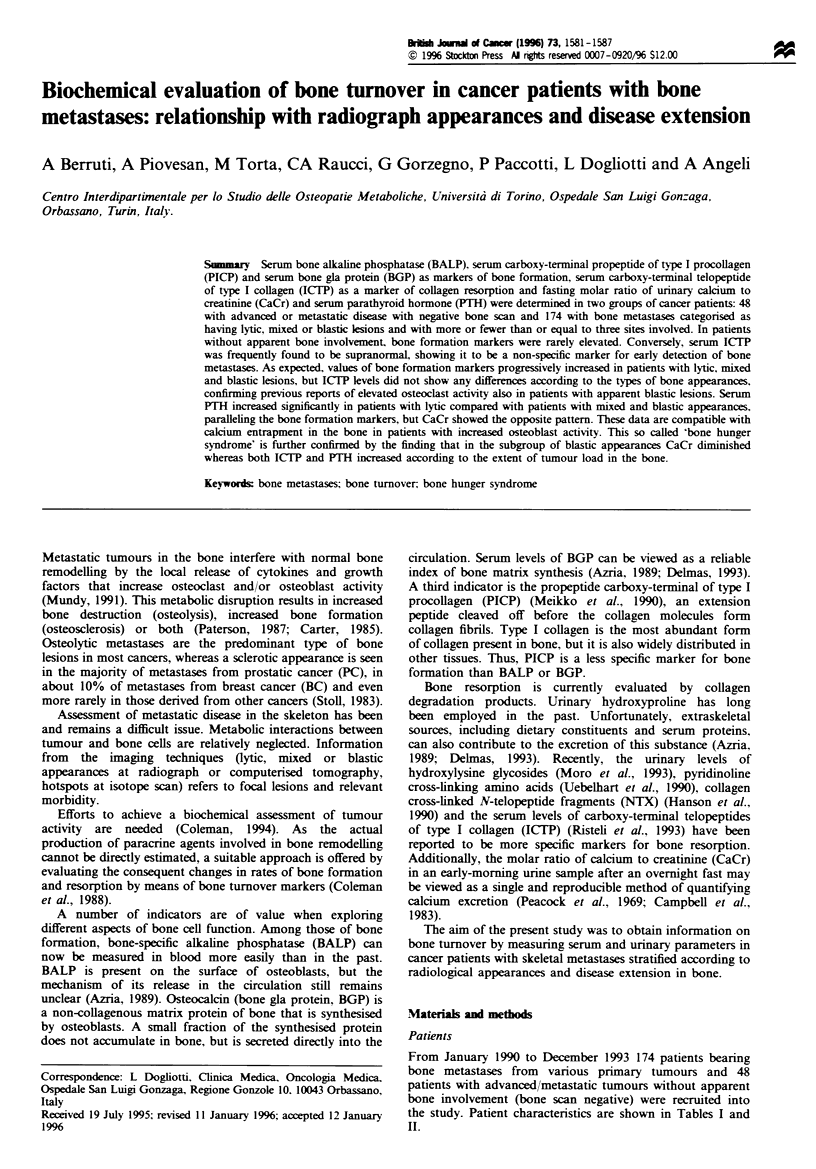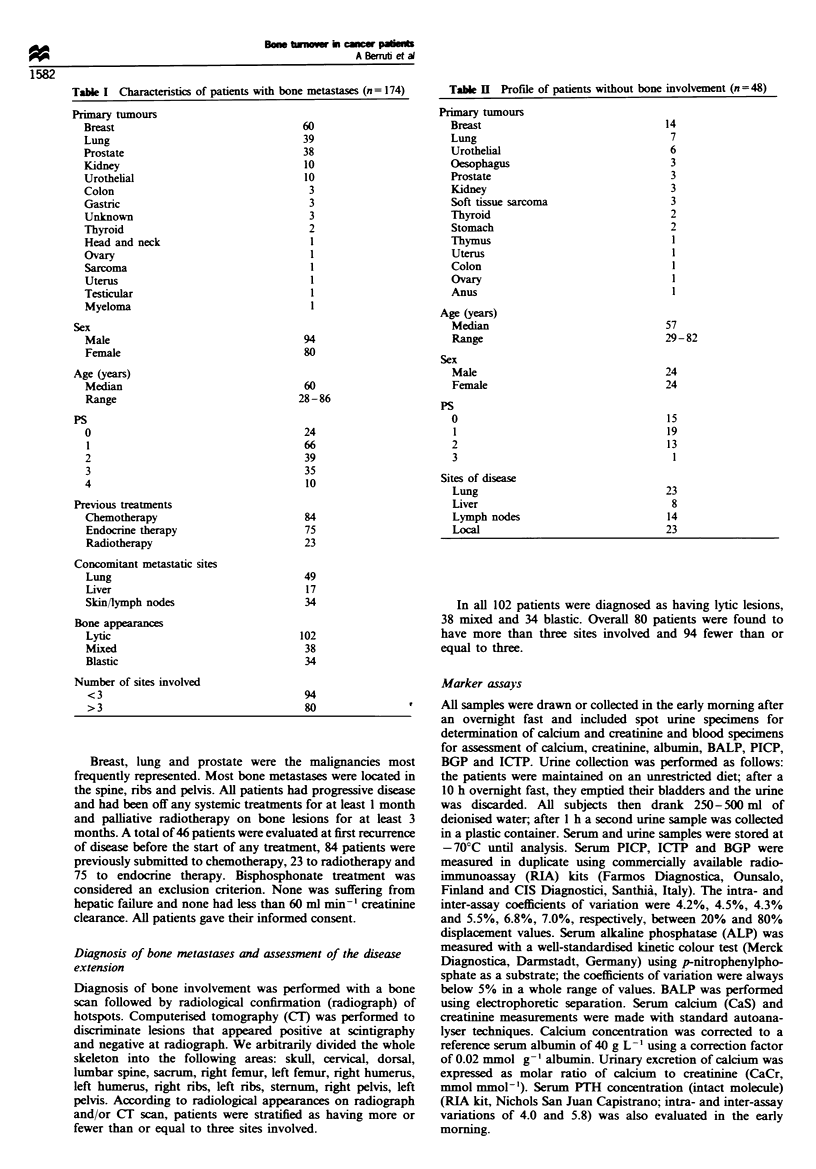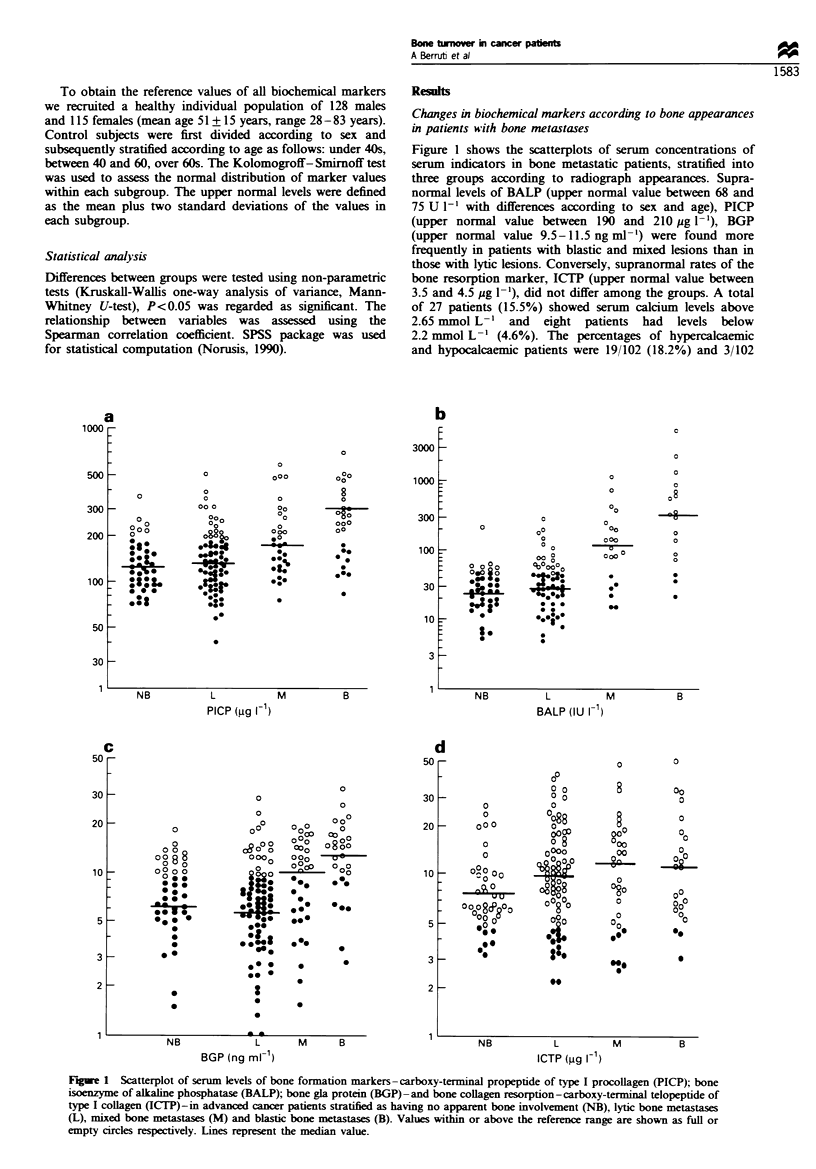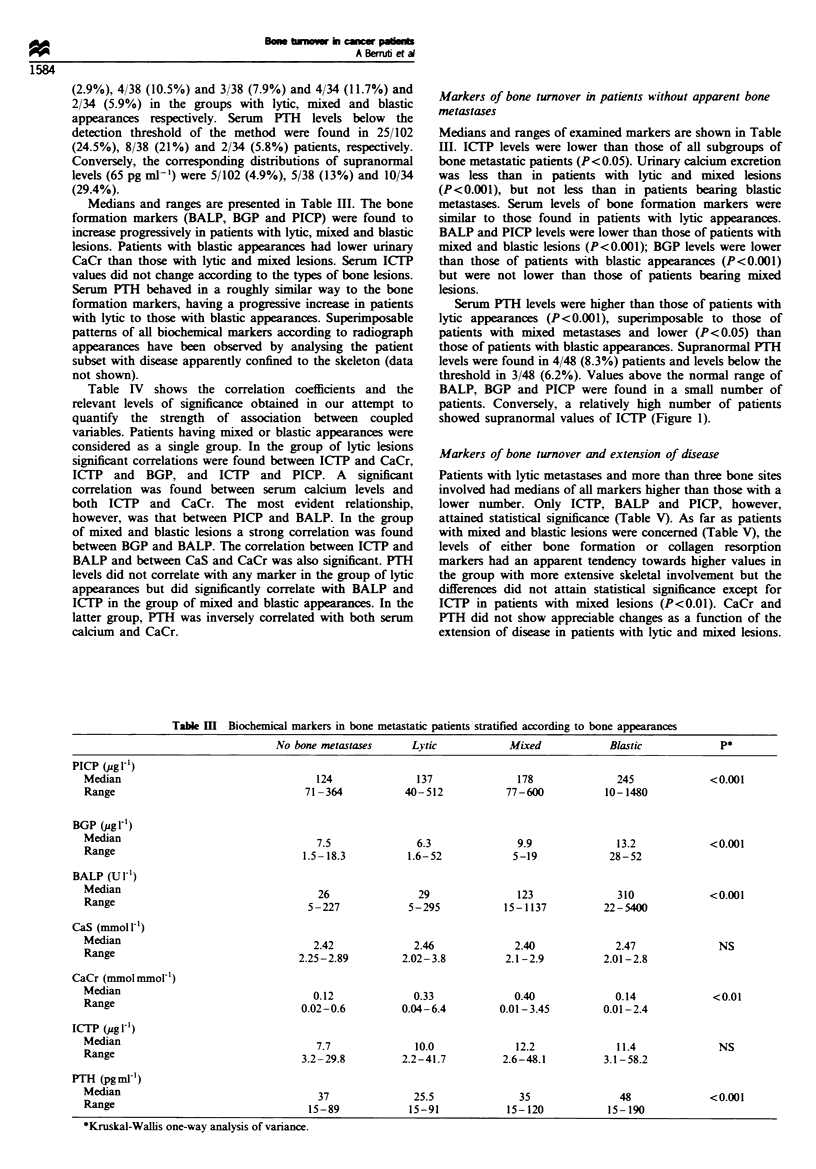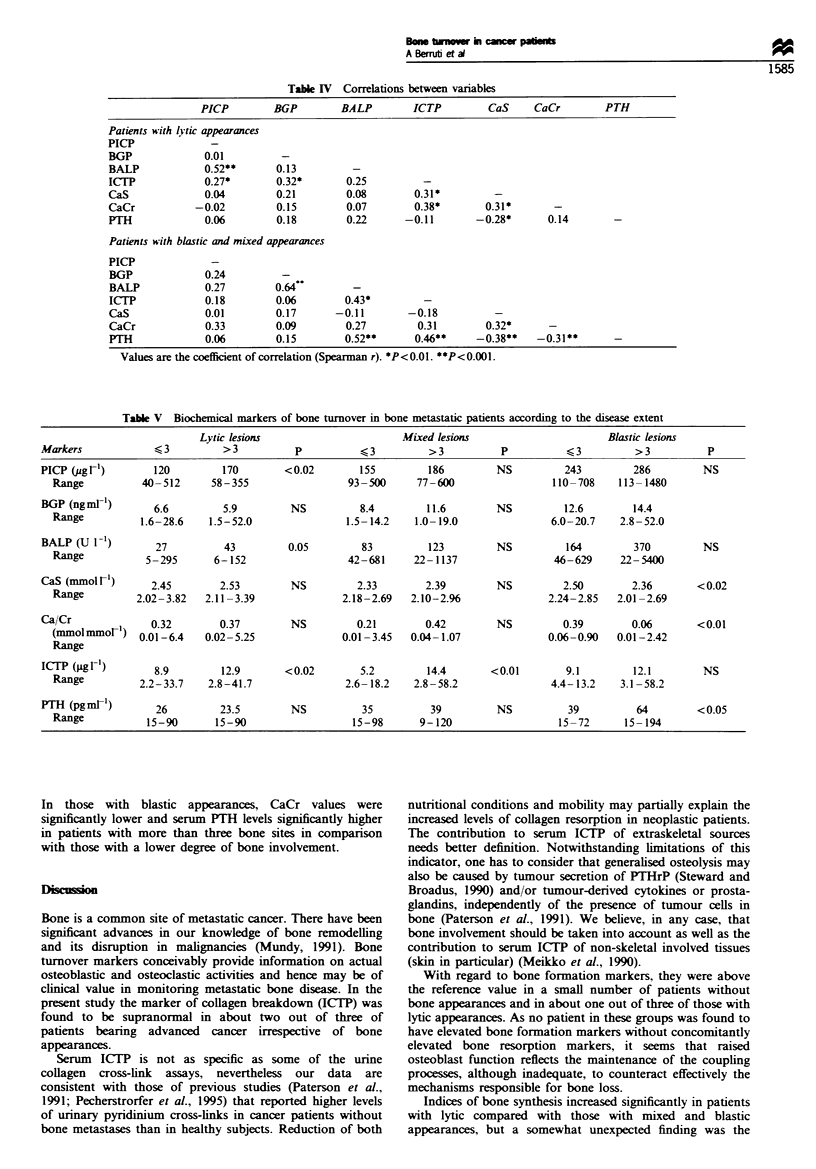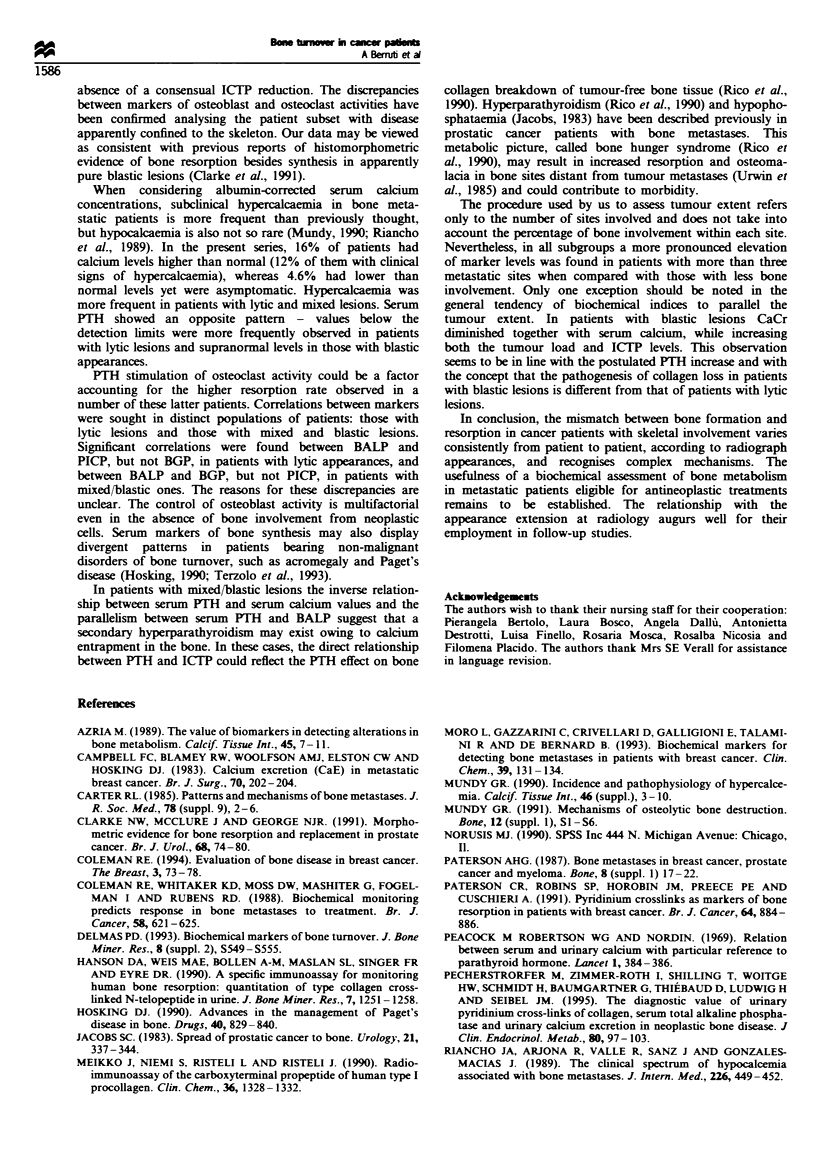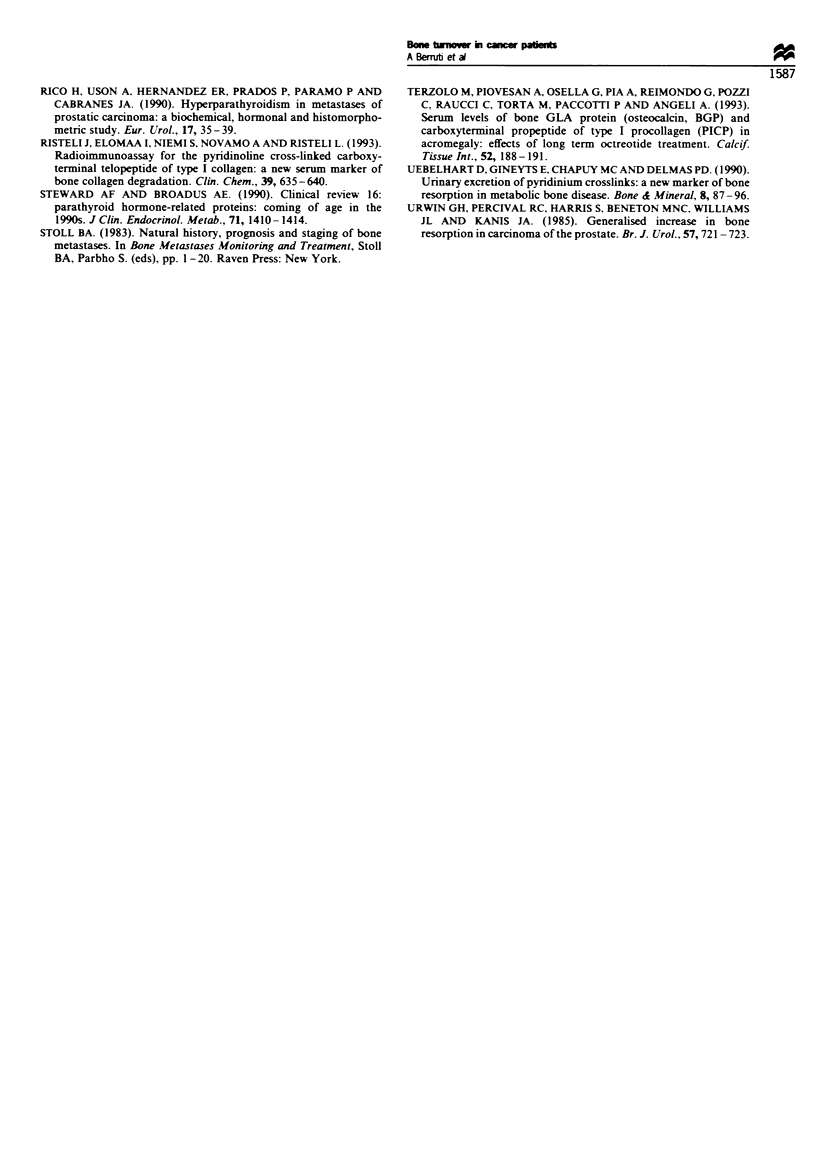# Biochemical evaluation of bone turnover in cancer patients with bone metastases: relationship with radiograph appearances and disease extension.

**DOI:** 10.1038/bjc.1996.298

**Published:** 1996-06

**Authors:** A. Berruti, A. Piovesan, M. Torta, C. A. Raucci, G. Gorzegno, P. Paccotti, L. Dogliotti, A. Angeli

**Affiliations:** Centro Interdipartimentale per lo Studio delle Osteopatie Metaboliche, Università di Torino, Ospedale San Luigi Gonzaga, Turin, Italy.

## Abstract

Serum bone alkaline phosphatase (BALP), serum carboxy-terminal propeptide of type I procollagen (PICP) and serum bone gla protein (BGP) as markers of bone formation, serum carboxy-terminal telopeptide of type I collagen (ICTP) as a marker of collagen resorption and fasting molar ratio of urinary calcium to creatinine (CaCr) and serum parathyroid hormone (PTH) were determined in two groups of cancer patients: 48 with advanced or metastatic disease with negative bone scan and 174 with bone metastases categorised as having lytic, mixed or blastic lesions and with more or fewer than or equal to three sites involved. In patients without apparent bone involvement, bone formation markers were rarely elevated. Conversely, serum ICTP was frequently found to be supranormal, showing it to be a non-specific marker for early detection of bone metastases. As expected, values of bone formation markers progressively increased in patients with lytic, mixed and blastic lesions, but ICTP levels did not show any differences according to the types of bone appearances, confirming previous reports of elevated osteoclast activity also in patients with apparent blastic lesions. Serum PTH increased significantly in patients with lytic compared with patients with mixed and blastic appearances, paralleling the bone formation markers, but CaCr showed the opposite pattern. These data are compatible with calcium entrapment in the bone in patients with increased osteoblast activity. This so called 'bone hunger syndrome' is further confirmed by the finding that in the subgroup of blastic appearances CaCr diminished whereas both ICTP and PTH increased according to the extent of tumour load in the bone.


British Journal of Cancer (1996) 73, 1581-1587

© 1996 Stockton Press All rights reserved 0007-0920/96 $12.00

### Biochemical evaluation of bone turnover in cancer patients with bone metastases: relationship with radiograph appearances and disease extension

A Berruti, A Piovesan, M Torta, CA Raucci, G Gorzegno, P Paccotti, L Dogliotti and A Angeli

*Centro Interdipartimentale per lo Studio delle Osteopatie Metaboliche, Università di Torino, Ospedale San Luigi Gonzaga, Orbassano, Turin, Italy.*

**Summary** Serum bone alkaline phosphatase (BALP), serum carboxy-terminal propeptide of type I procollagen (PICP) and serum bone gla protein (BGP) as markers of bone formation, serum carboxy-terminal telopeptide of type I collagen (ICTP) as a marker of collagen resorption and fasting molar ratio of urinary calcium to creatinine (CaCr) and serum parathyroid hormone (PTH) were determined in two groups of cancer patients: 48 with advanced or metastatic disease with negative bone scan and 174 with bone metastases categorised as having lytic, mixed or blastic lesions and with more or fewer than or equal to three sites involved. In patients without apparent bone involvement, bone formation markers were rarely elevated. Conversely, serum ICTP was frequently found to be supranormal, showing it to be a non-specific marker for early detection of bone metastases. As expected, values of bone formation markers progressively increased in patients with lytic, mixed and blastic lesions, but ICTP levels did not show any differences according to the types of bone appearances, confirming previous reports of elevated osteoclast activity also in patients with apparent blastic lesions. Serum PTH increased significantly in patients with lytic compared with patients with mixed and blastic appearances, paralleling the bone formation markers, but CaCr showed the opposite pattern. These data are compatible with calcium entrapment in the bone in patients with increased osteoblast activity. This so called 'bone hunger syndrome' is further confirmed by the finding that in the subgroup of blastic appearances CaCr diminished whereas both ICTP and PTH increased according to the extent of tumour load in the bone.

**Keywords:** bone metastases; bone turnover; bone hunger syndrome

Metastatic tumours in the bone interfere with normal bone remodelling by the local release of cytokines and growth factors that increase osteoclast and/or osteoblast activity (Mundy, 1991). This metabolic disruption results in increased bone destruction (osteolysis), increased bone formation (osteosclerosis) or both (Paterson, 1987; Carter, 1985). Osteolytic metastases are the predominant type of bone lesions in most cancers, whereas a sclerotic appearance is seen in the majority of metastases from prostatic cancer (PC), in about 10% of metastases from breast cancer (BC) and even more rarely in those derived from other cancers (Stoll, 1983).

Assessment of metastatic disease in the skeleton has been and remains a difficult issue. Metabolic interactions between tumour and bone cells are relatively neglected. Information from the imaging techniques (lytic, mixed or blastic appearances at radiograph or computerised tomography, hotspots at isotope scan) refers to focal lesions and relevant morbidity.

Efforts to achieve a biochemical assessment of tumour activity are needed (Coleman, 1994). As the actual production of paracrine agents involved in bone remodelling cannot be directly estimated, a suitable approach is offered by evaluating the consequent changes in rates of bone formation and resorption by means of bone turnover markers (Coleman *et al.*, 1988).

A number of indicators are of value when exploring different aspects of bone cell function. Among those of bone formation, bone-specific alkaline phosphatase (BALP) can now be measured in blood more easily than in the past. BALP is present on the surface of osteoblasts, but the mechanism of its release in the circulation still remains unclear (Azria, 1989). Osteocalcin (bone gla protein, BGP) is a non-collagenous matrix protein of bone that is synthesised by osteoblasts. A small fraction of the synthesised protein does not accumulate in bone, but is secreted directly into the circulation. Serum levels of BGP can be viewed as a reliable index of bone matrix synthesis (Azria, 1989; Delmas, 1993). A third indicator is the propeptide carboxy-terminal of type I procollagen (PICP) (Meikko *et al.*, 1990), an extension peptide cleaved off before the collagen molecules form collagen fibrils. Type I collagen is the most abundant form of collagen present in bone, but it is also widely distributed in other tissues. Thus, PICP is a less specific marker for bone formation than BALP or BGP.

Bone resorption is currently evaluated by collagen degradation products. Urinary hydroxyproline has long been employed in the past. Unfortunately, extraskeletal sources, including dietary constituents and serum proteins, can also contribute to the excretion of this substance (Azria, 1989; Delmas, 1993). Recently, the urinary levels of hydroxylysine glycosides (Moro *et al.*, 1993), pyridinoline cross-linking amino acids (Uebelhart *et al.*, 1990), collagen cross-linked *N*-telopeptide fragments (NTX) (Hanson *et al.*, 1990) and the serum levels of carboxy-terminal telopeptides of type I collagen (ICTP) (Risteli *et al.*, 1993) have been reported to be more specific markers for bone resorption. Additionally, the molar ratio of calcium to creatinine (CaCr) in an early-morning urine sample after an overnight fast may be viewed as a single and reproducible method of quantifying calcium excretion (Peacock *et al.*, 1969; Campbell *et al.*, 1983).

The aim of the present study was to obtain information on bone turnover by measuring serum and urinary parameters in cancer patients with skeletal metastases stratified according to radiological appearances and disease extension in bone.

### Materials and methods

### *Patients*

From January 1990 to December 1993 174 patients bearing bone metastases from various primary tumours and 48 patients with advanced/metastatic tumours without apparent bone involvement (bone scan negative) were recruited into the study. Patient characteristics are shown in Tables I and II.

Correspondence: L Dogliotti, Clinica Medica, Oncologia Medica, Ospedale San Luigi Gonzaga, Regione Gonzole 10, 10043 Orbassano, Italy
Received 19 July 1995; revised 11 January 1996; accepted 12 January 1996

**Table I** Characteristics of patients with bone metastases ($n = 174$)

| | |
|---|---|
| Primary tumours | |
| Breast | 60 |
| Lung | 39 |
| Prostate | 38 |
| Kidney | 10 |
| Urothelial | 10 |
| Colon | 3 |
| Gastric | 3 |
| Unknown | 3 |
| Thyroid | 2 |
| Head and neck | 1 |
| Ovary | 1 |
| Sarcoma | 1 |
| Uterus | 1 |
| Testicular | 1 |
| Myeloma | 1 |
| Sex | |
| Male | 94 |
| Female | 80 |
| Age (years) | |
| Median | 60 |
| Range | 28-86 |
| PS | |
| 0 | 24 |
| 1 | 66 |
| 2 | 39 |
| 3 | 35 |
| 4 | 10 |
| Previous treatments | |
| Chemotherapy | 84 |
| Endocrine therapy | 75 |
| Radiotherapy | 23 |
| Concomitant metastatic sites | |
| Lung | 49 |
| Liver | 17 |
| Skin/lymph nodes | 34 |
| Bone appearances | |
| Lytic | 102 |
| Mixed | 38 |
| Blastic | 34 |
| Number of sites involved | |
| $<3$ | 94 |
| $>3$ | 80 |

Breast, lung and prostate were the malignancies most frequently represented. Most bone metastases were located in the spine, ribs and pelvis. All patients had progressive disease and had been off any systemic treatments for at least 1 month and palliative radiotherapy on bone lesions for at least 3 months. A total of 46 patients were evaluated at first recurrence of disease before the start of any treatment, 84 patients were previously submitted to chemotherapy, 23 to radiotherapy and 75 to endocrine therapy. Bisphosphonate treatment was considered an exclusion criterion. None was suffering from hepatic failure and none had less than 60 ml $min^{-1}$ creatinine clearance. All patients gave their informed consent.

### *Diagnosis of bone metastases and assessment of the disease extension*

Diagnosis of bone involvement was performed with a bone scan followed by radiological confirmation (radiograph) of hotspots. Computerised tomography (CT) was performed to discriminate lesions that appeared positive at scintigraphy and negative at radiograph. We arbitrarily divided the whole skeleton into the following areas: skull, cervical, dorsal, lumbar spine, sacrum, right femur, left femur, right humerus, left humerus, right ribs, left ribs, sternum, right pelvis, left pelvis. According to radiological appearances on radiograph and/or CT scan, patients were stratified as having more or fewer than or equal to three sites involved.

**Table II** Profile of patients without bone involvement ($n = 48$)

| | |
|---|---|
| Primary tumours | |
| Breast | 14 |
| Lung | 7 |
| Urothelial | 6 |
| Oesophagus | 3 |
| Prostate | 3 |
| Kidney | 3 |
| Soft tissue sarcoma | 3 |
| Thyroid | 2 |
| Stomach | 2 |
| Thymus | 1 |
| Uterus | 1 |
| Colon | 1 |
| Ovary | 1 |
| Anus | 1 |
| Age (years) | |
| Median | 57 |
| Range | 29-82 |
| Sex | |
| Male | 24 |
| Female | 24 |
| PS | |
| 0 | 15 |
| 1 | 19 |
| 2 | 13 |
| 3 | 1 |
| Sites of disease | |
| Lung | 23 |
| Liver | 8 |
| Lymph nodes | 14 |
| Local | 23 |

In all 102 patients were diagnosed as having lytic lesions, 38 mixed and 34 blastic. Overall 80 patients were found to have more than three sites involved and 94 fewer than or equal to three.

### *Marker assays*

All samples were drawn or collected in the early morning after an overnight fast and included spot urine specimens for determination of calcium and creatinine and blood specimens for assessment of calcium, creatinine, albumin, BALP, PICP, BGP and ICTP. Urine collection was performed as follows: the patients were maintained on an unrestricted diet; after a 10 h overnight fast, they emptied their bladders and the urine was discarded. All subjects then drank 250-500 ml of deionised water; after 1 h a second urine sample was collected in a plastic container. Serum and urine samples were stored at $-70°C$ until analysis. Serum PICP, ICTP and BGP were measured in duplicate using commercially available radioimmunoassay (RIA) kits (Farmos Diagnostica, Ounsalo, Finland and CIS Diagnostici, Santhià, Italy). The intra- and inter-assay coefficients of variation were 4.2%, 4.5%, 4.3% and 5.5%, 6.8%, 7.0%, respectively, between 20% and 80% displacement values. Serum alkaline phosphatase (ALP) was measured with a well-standardised kinetic colour test (Merck Diagnostica, Darmstadt, Germany) using *p*-nitrophenylphosphate as a substrate; the coefficients of variation were always below 5% in a whole range of values. BALP was performed using electrophoretic separation. Serum calcium (CaS) and creatinine measurements were made with standard autoanalyser techniques. Calcium concentration was corrected to a reference serum albumin of 40 g $L^{-1}$ using a correction factor of 0.02 mmol $g^{-1}$ albumin. Urinary excretion of calcium was expressed as molar ratio of calcium to creatinine (CaCr, mmol $mmol^{-1}$). Serum PTH concentration (intact molecule) (RIA kit, Nichols San Juan Capistrano; intra- and inter-assay variations of 4.0 and 5.8) was also evaluated in the early morning.

To obtain the reference values of all biochemical markers we recruited a healthy individual population of 128 males and 115 females (mean age 51 ± 15 years, range 28–83 years). Control subjects were first divided according to sex and subsequently stratified according to age as follows: under 40s, between 40 and 60, over 60s. The Kolomogroff–Smirnoff test was used to assess the normal distribution of marker values within each subgroup. The upper normal levels were defined as the mean plus two standard deviations of the values in each subgroup.

### Statistical analysis

Differences between groups were tested using non-parametric tests (Kruskall-Wallis one-way analysis of variance, Mann-Whitney *U*-test), $P<0.05$ was regarded as significant. The relationship between variables was assessed using the Spearman correlation coefficient. SPSS package was used for statistical computation (Norusis, 1990).

### Results

### Changes in biochemical markers according to bone appearances in patients with bone metastases

Figure 1 shows the scatterplots of serum concentrations of serum indicators in bone metastatic patients, stratified into three groups according to radiograph appearances. Supranormal levels of BALP (upper normal value between 68 and 75 U $l^{-1}$ with differences according to sex and age), PICP (upper normal value between 190 and 210 $\mu g\ l^{-1}$), BGP (upper normal value 9.5–11.5 ng $ml^{-1}$) were found more frequently in patients with blastic and mixed lesions than in those with lytic lesions. Conversely, supranormal rates of the bone resorption marker, ICTP (upper normal value between 3.5 and 4.5 $\mu g\ l^{-1}$), did not differ among the groups. A total of 27 patients (15.5%) showed serum calcium levels above 2.65 mmol $L^{-1}$ and eight patients had levels below 2.2 mmol $L^{-1}$ (4.6%). The percentages of hypercalcaemic and hypocalcaemic patients were 19/102 (18.2%) and 3/102

**Figure 1** Scatterplot of serum levels of bone formation markers–carboxy-terminal propeptide of type I procollagen (PICP); bone isoenzyme of alkaline phosphatase (BALP); bone gla protein (BGP)–and bone collagen resorption–carboxy-terminal telopeptide of type I collagen (ICTP)–in advanced cancer patients stratified as having no apparent bone involvement (NB), lytic bone metastases (L), mixed bone metastases (M) and blastic bone metastases (B). Values within or above the reference range are shown as full or empty circles respectively. Lines represent the median value.

(2.9%), 4/38 (10.5%) and 3/38 (7.9%) and 4/34 (11.7%) and 2/34 (5.9%) in the groups with lytic, mixed and blastic appearances respectively. Serum PTH levels below the detection threshold of the method were found in 25/102 (24.5%), 8/38 (21%) and 2/34 (5.8%) patients, respectively. Conversely, the corresponding distributions of supranormal levels (65 pg $ml^{-1}$) were 5/102 (4.9%), 5/38 (13%) and 10/34 (29.4%).

Medians and ranges are presented in Table III. The bone formation markers (BALP, BGP and PICP) were found to increase progressively in patients with lytic, mixed and blastic lesions. Patients with blastic appearances had lower urinary CaCr than those with lytic and mixed lesions. Serum ICTP values did not change according to the types of bone lesions. Serum PTH behaved in a roughly similar way to the bone formation markers, having a progressive increase in patients with lytic to those with blastic appearances. Superimposable patterns of all biochemical markers according to radiograph appearances have been observed by analysing the patient subset with disease apparently confined to the skeleton (data not shown).

Table IV shows the correlation coefficients and the relevant levels of significance obtained in our attempt to quantify the strength of association between coupled variables. Patients having mixed or blastic appearances were considered as a single group. In the group of lytic lesions significant correlations were found between ICTP and CaCr, ICTP and BGP, and ICTP and PICP. A significant correlation was found between serum calcium levels and both ICTP and CaCr. The most evident relationship, however, was that between PICP and BALP. In the group of mixed and blastic lesions a strong correlation was found between BGP and BALP. The correlation between ICTP and BALP and between CaS and CaCr was also significant. PTH levels did not correlate with any marker in the group of lytic appearances but did significantly correlate with BALP and ICTP in the group of mixed and blastic appearances. In the latter group, PTH was inversely correlated with both serum calcium and CaCr.

### *Markers of bone turnover in patients without apparent bone metastases*

Medians and ranges of examined markers are shown in Table III. ICTP levels were lower than those of all subgroups of bone metastatic patients ($P<0.05$). Urinary calcium excretion was less than in patients with lytic and mixed lesions ($P<0.001$), but not less than in patients bearing blastic metastases. Serum levels of bone formation markers were similar to those found in patients with lytic appearances. BALP and PICP levels were lower than those of patients with mixed and blastic lesions ($P<0.001$); BGP levels were lower than those of patients with blastic appearances ($P<0.001$) but were not lower than those of patients bearing mixed lesions.

Serum PTH levels were higher than those of patients with lytic appearances ($P<0.001$), superimposable to those of patients with mixed metastases and lower ($P<0.05$) than those of patients with blastic appearances. Supranormal PTH levels were found in 4/48 (8.3%) patients and levels below the threshold in 3/48 (6.2%). Values above the normal range of BALP, BGP and PICP were found in a small number of patients. Conversely, a relatively high number of patients showed supranormal values of ICTP (Figure 1).

### *Markers of bone turnover and extension of disease*

Patients with lytic metastases and more than three bone sites involved had medians of all markers higher than those with a lower number. Only ICTP, BALP and PICP, however, attained statistical significance (Table V). As far as patients with mixed and blastic lesions were concerned (Table V), the levels of either bone formation or collagen resorption markers had an apparent tendency towards higher values in the group with more extensive skeletal involvement but the differences did not attain statistical significance except for ICTP in patients with mixed lesions ($P<0.01$). CaCr and PTH did not show appreciable changes as a function of the extension of disease in patients with lytic and mixed lesions.

**Table III** Biochemical markers in bone metastatic patients stratified according to bone appearances

| | *No bone metastases* | *Lytic* | *Mixed* | *Blastic* | P* |
|---|---|---|---|---|---|
| PICP ($\mu g\,l^{-1}$) | | | | | |
| Median | 124 | 137 | 178 | 245 | <0.001 |
| Range | 71-364 | 40-512 | 77-600 | 10-1480 | |
| BGP ($\mu g\,l^{-1}$) | | | | | |
| Median | 7.5 | 6.3 | 9.9 | 13.2 | <0.001 |
| Range | 1.5-18.3 | 1.6-52 | 5-19 | 28-52 | |
| BALP ($U\,l^{-1}$) | | | | | |
| Median | 26 | 29 | 123 | 310 | <0.001 |
| Range | 5-227 | 5-295 | 15-1137 | 22-5400 | |
| CaS ($mmol\,l^{-1}$) | | | | | |
| Median | 2.42 | 2.46 | 2.40 | 2.47 | NS |
| Range | 2.25-2.89 | 2.02-3.8 | 2.1-2.9 | 2.01-2.8 | |
| CaCr ($mmol\,mmol^{-1}$) | | | | | |
| Median | 0.12 | 0.33 | 0.40 | 0.14 | <0.01 |
| Range | 0.02-0.6 | 0.04-6.4 | 0.01-3.45 | 0.01-2.4 | |
| ICTP ($\mu g\,l^{-1}$) | | | | | |
| Median | 7.7 | 10.0 | 12.2 | 11.4 | NS |
| Range | 3.2-29.8 | 2.2-41.7 | 2.6-48.1 | 3.1-58.2 | |
| PTH ($pg\,ml^{-1}$) | | | | | |
| Median | 37 | 25.5 | 35 | 48 | <0.001 |
| Range | 15-89 | 15-91 | 15-120 | 15-190 | |

*Kruskal-Wallis one-way analysis of variance.

Table IV Correlations between variables

| | PICP | BGP | BALP | ICTP | CaS | CaCr | PTH |
|---|---|---|---|---|---|---|---|
| *Patients with lytic appearances* | | | | | | | |
| PICP | – | | | | | | |
| BGP | 0.01 | – | | | | | |
| BALP | 0.52** | 0.13 | – | | | | |
| ICTP | 0.27* | 0.32* | 0.25 | – | | | |
| CaS | 0.04 | 0.21 | 0.08 | 0.31* | – | | |
| CaCr | −0.02 | 0.15 | 0.07 | 0.38* | 0.31* | – | |
| PTH | 0.06 | 0.18 | 0.22 | −0.11 | −0.28* | 0.14 | – |
| *Patients with blastic and mixed appearances* | | | | | | | |
| PICP | – | | | | | | |
| BGP | 0.24 | – | | | | | |
| BALP | 0.27 | 0.64** | – | | | | |
| ICTP | 0.18 | 0.06 | 0.43* | – | | | |
| CaS | 0.01 | 0.17 | −0.11 | −0.18 | – | | |
| CaCr | 0.33 | 0.09 | 0.27 | 0.31 | 0.32* | – | |
| PTH | 0.06 | 0.15 | 0.52** | 0.46** | −0.38** | −0.31** | – |

Values are the coefficient of correlation (Spearman $r$). $^*P<0.01$. $^{**}P<0.001$.

Table V Biochemical markers of bone turnover in bone metastatic patients according to the disease extent

| Markers | Lytic lesions ≤3 | Lytic lesions >3 | P | Mixed lesions ≤3 | Mixed lesions >3 | P | Blastic lesions ≤3 | Blastic lesions >3 | P |
|---|---|---|---|---|---|---|---|---|---|
| PICP ($\mu g\,l^{-1}$) | 120 | 170 | <0.02 | 155 | 186 | NS | 243 | 286 | NS |
| Range | 40–512 | 58–355 | | 93–500 | 77–600 | | 110–708 | 113–1480 | |
| BGP ($ng\,ml^{-1}$) | 6.6 | 5.9 | NS | 8.4 | 11.6 | NS | 12.6 | 14.4 | |
| Range | 1.6–28.6 | 1.5–52.0 | | 1.5–14.2 | 1.0–19.0 | | 6.0–20.7 | 2.8–52.0 | |
| BALP ($U\,l^{-1}$) | 27 | 43 | 0.05 | 83 | 123 | NS | 164 | 370 | NS |
| Range | 5–295 | 6–152 | | 42–681 | 22–1137 | | 46–629 | 22–5400 | |
| CaS ($mmol\,l^{-1}$) | 2.45 | 2.53 | NS | 2.33 | 2.39 | NS | 2.50 | 2.36 | <0.02 |
| Range | 2.02–3.82 | 2.11–3.39 | | 2.18–2.69 | 2.10–2.96 | | 2.24–2.85 | 2.01–2.69 | |
| Ca/Cr ($mmol\,mmol^{-1}$) | 0.32 | 0.37 | NS | 0.21 | 0.42 | NS | 0.39 | 0.06 | <0.01 |
| Range | 0.01–6.4 | 0.02–5.25 | | 0.01–3.45 | 0.04–1.07 | | 0.06–0.90 | 0.01–2.42 | |
| ICTP ($\mu g\,l^{-1}$) | 8.9 | 12.9 | <0.02 | 5.2 | 14.4 | <0.01 | 9.1 | 12.1 | NS |
| Range | 2.2–33.7 | 2.8–41.7 | | 2.6–18.2 | 2.8–58.2 | | 4.4–13.2 | 3.1–58.2 | |
| PTH ($pg\,ml^{-1}$) | 26 | 23.5 | NS | 35 | 39 | NS | 39 | 64 | <0.05 |
| Range | 15–90 | 15–90 | | 15–98 | 9–120 | | 15–72 | 15–194 | |

In those with blastic appearances, CaCr values were significantly lower and serum PTH levels significantly higher in patients with more than three bone sites in comparison with those with a lower degree of bone involvement.

### Discussion

Bone is a common site of metastatic cancer. There have been significant advances in our knowledge of bone remodelling and its disruption in malignancies (Mundy, 1991). Bone turnover markers conceivably provide information on actual osteoblastic and osteoclastic activities and hence may be of clinical value in monitoring metastatic bone disease. In the present study the marker of collagen breakdown (ICTP) was found to be supranormal in about two out of three of patients bearing advanced cancer irrespective of bone appearances.

Serum ICTP is not as specific as some of the urine collagen cross-link assays, nevertheless our data are consistent with those of previous studies (Paterson *et al.*, 1991; Pecherstrorfer *et al.*, 1995) that reported higher levels of urinary pyridinium cross-links in cancer patients without bone metastases than in healthy subjects. Reduction of both nutritional conditions and mobility may partially explain the increased levels of collagen resorption in neoplastic patients. The contribution to serum ICTP of extraskeletal sources needs better definition. Notwithstanding limitations of this indicator, one has to consider that generalised osteolysis may also be caused by tumour secretion of PTHrP (Steward and Broadus, 1990) and/or tumour-derived cytokines or prostaglandins, independently of the presence of tumour cells in bone (Paterson *et al.*, 1991). We believe, in any case, that bone involvement should be taken into account as well as the contribution to serum ICTP of non-skeletal involved tissues (skin in particular) (Meikko *et al.*, 1990).

With regard to bone formation markers, they were above the reference value in a small number of patients without bone appearances and in about one out of three of those with lytic appearances. As no patient in these groups was found to have elevated bone formation markers without concomitantly elevated bone resorption markers, it seems that raised osteoblast function reflects the maintenance of the coupling processes, although inadequate, to counteract effectively the mechanisms responsible for bone loss.

Indices of bone synthesis increased significantly in patients with lytic compared with those with mixed and blastic appearances, but a somewhat unexpected finding was the absence of a consensual ICTP reduction. The discrepancies between markers of osteoblast and osteoclast activities have been confirmed analysing the patient subset with disease apparently confined to the skeleton. Our data may be viewed as consistent with previous reports of histomorphometric evidence of bone resorption besides synthesis in apparently pure blastic lesions (Clarke *et al.*, 1991).

When considering albumin-corrected serum calcium concentrations, subclinical hypercalcaemia in bone metastatic patients is more frequent than previously thought, but hypocalcaemia is also not so rare (Mundy, 1990; Riancho *et al.*, 1989). In the present series, 16% of patients had calcium levels higher than normal (12% of them with clinical signs of hypercalcaemia), whereas 4.6% had lower than normal levels yet were asymptomatic. Hypercalcaemia was more frequent in patients with lytic and mixed lesions. Serum PTH showed an opposite pattern – values below the detection limits were more frequently observed in patients with lytic lesions and supranormal levels in those with blastic appearances.

PTH stimulation of osteoclast activity could be a factor accounting for the higher resorption rate observed in a number of these latter patients. Correlations between markers were sought in distinct populations of patients: those with lytic lesions and those with mixed and blastic lesions. Significant correlations were found between BALP and PICP, but not BGP, in patients with lytic appearances, and between BALP and BGP, but not PICP, in patients with mixed/blastic ones. The reasons for these discrepancies are unclear. The control of osteoblast activity is multifactorial even in the absence of bone involvement from neoplastic cells. Serum markers of bone synthesis may also display divergent patterns in patients bearing non-malignant disorders of bone turnover, such as acromegaly and Paget's disease (Hosking, 1990; Terzolo *et al.*, 1993).

In patients with mixed/blastic lesions the inverse relationship between serum PTH and serum calcium values and the parallelism between serum PTH and BALP suggest that a secondary hyperparathyroidism may exist owing to calcium entrapment in the bone. In these cases, the direct relationship between PTH and ICTP could reflect the PTH effect on bone collagen breakdown of tumour-free bone tissue (Rico *et al.*, 1990). Hyperparathyroidism (Rico *et al.*, 1990) and hypophosphataemia (Jacobs, 1983) have been described previously in prostatic cancer patients with bone metastases. This metabolic picture, called bone hunger syndrome (Rico *et al.*, 1990), may result in increased resorption and osteomalacia in bone sites distant from tumour metastases (Urwin *et al.*, 1985) and could contribute to morbidity.

The procedure used by us to assess tumour extent refers only to the number of sites involved and does not take into account the percentage of bone involvement within each site. Nevertheless, in all subgroups a more pronounced elevation of marker levels was found in patients with more than three metastatic sites when compared with those with less bone involvement. Only one exception should be noted in the general tendency of biochemical indices to parallel the tumour extent. In patients with blastic lesions CaCr diminished together with serum calcium, while increasing both the tumour load and ICTP levels. This observation seems to be in line with the postulated PTH increase and with the concept that the pathogenesis of collagen loss in patients with blastic lesions is different from that of patients with lytic lesions.

In conclusion, the mismatch between bone formation and resorption in cancer patients with skeletal involvement varies consistently from patient to patient, according to radiograph appearances, and recognises complex mechanisms. The usefulness of a biochemical assessment of bone metabolism in metastatic patients eligible for antineoplastic treatments remains to be established. The relationship with the appearance extension at radiology augurs well for their employment in follow-up studies.

**Acknowledgements**
The authors wish to thank their nursing staff for their cooperation: Pierangela Bertolo, Laura Bosco, Angela Dallù, Antonietta Destrotti, Luisa Finello, Rosaria Mosca, Rosalba Nicosia and Filomena Placido. The authors thank Mrs SE Verall for assistance in language revision.

### References

AZRIA M. (1989). The value of biomarkers in detecting alterations in bone metabolism. *Calcif. Tissue Int.*, **45**, 7–11.

CAMPBELL FC, BLAMEY RW, WOOLFSON AMJ, ELSTON CW AND HOSKING DJ. (1983). Calcium excretion (CaE) in metastatic breast cancer. *Br. J. Surg.*, **70**, 202–204.

CARTER RL. (1985). Patterns and mechanisms of bone metastases. *J. R. Soc. Med.*, **78** (suppl. 9), 2–6.

CLARKE NW, MCCLURE J AND GEORGE NJR. (1991). Morphometric evidence for bone resorption and replacement in prostate cancer. *Br. J. Urol.*, **68**, 74–80.

COLEMAN RE. (1994). Evaluation of bone disease in breast cancer. *The Breast*, **3**, 73–78.

COLEMAN RE, WHITAKER KD, MOSS DW, MASHITER G, FOGELMAN I AND RUBENS RD. (1988). Biochemical monitoring predicts response in bone metastases to treatment. *Br. J. Cancer*, **58**, 621–625.

DELMAS PD. (1993). Biochemical markers of bone turnover. *J. Bone Miner. Res.*, **8** (suppl. 2), S549–S555.

HANSON DA, WEIS MAE, BOLLEN A-M, MASLAN SL, SINGER FR AND EYRE DR. (1990). A specific immunoassay for monitoring human bone resorption: quantitation of type collagen cross-linked N-telopeptide in urine. *J. Bone Miner. Res.*, **7**, 1251–1258.

HOSKING DJ. (1990). Advances in the management of Paget's disease in bone. *Drugs*, **40**, 829–840.

JACOBS SC. (1983). Spread of prostatic cancer to bone. *Urology*, **21**, 337–344.

MEIKKO J, NIEMI S, RISTELI L AND RISTELI J. (1990). Radioimmunoassay of the carboxyterminal propeptide of human type I procollagen. *Clin. Chem.*, **36**, 1328–1332.

MORO L, GAZZARINI C, CRIVELLARI D, GALLIGIONI E, TALAMINI R AND DE BERNARD B. (1993). Biochemical markers for detecting bone metastases in patients with breast cancer. *Clin. Chem.*, **39**, 131–134.

MUNDY GR. (1990). Incidence and pathophysiology of hypercalcemia. *Calcif. Tissue Int.*, **46** (suppl.), 3–10.

MUNDY GR. (1991). Mechanisms of osteolytic bone destruction. *Bone*, **12** (suppl. 1), S1–S6.

NORUSIS MJ. (1990). SPSS Inc 444 N. Michigan Avenue: Chicago, Il.

PATERSON AHG. (1987). Bone metastases in breast cancer, prostate cancer and myeloma. *Bone*, **8** (suppl. 1) 17–22.

PATERSON CR, ROBINS SP, HOROBIN JM, PREECE PE AND CUSCHIERI A. (1991). Pyridinium crosslinks as markers of bone resorption in patients with breast cancer. *Br. J. Cancer*, **64**, 884–886.

PEACOCK M ROBERTSON WG AND NORDIN. (1969). Relation between serum and urinary calcium with particular reference to parathyroid hormone. *Lancet* **1**, 384–386.

PECHERSTRORFER M, ZIMMER-ROTH I, SHILLING T, WOITGE HW, SCHMIDT H, BAUMGARTNER G, THIÉBAUD D, LUDWIG H AND SEIBEL JM. (1995). The diagnostic value of urinary pyridinium cross-links of collagen, serum total alkaline phosphatase and urinary calcium excretion in neoplastic bone disease. *J Clin. Endocrinol. Metab.*, **80**, 97–103.

RIANCHO JA, ARJONA R, VALLE R, SANZ J AND GONZALES-MACIAS J. (1989). The clinical spectrum of hypocalcemia associated with bone metastases. *J. Intern. Med.*, **226**, 449–452.

RICO H, USON A, HERNANDEZ ER, PRADOS P, PARAMO P AND CABRANES JA. (1990). Hyperparathyroidism in metastases of prostatic carcinoma: a biochemical, hormonal and histomorphometric study. *Eur. Urol.*, **17**, 35–39.

RISTELI J, ELOMAA I, NIEMI S, NOVAMO A AND RISTELI L. (1993). Radioimmunoassay for the pyridinoline cross-linked carboxy-terminal telopeptide of type I collagen: a new serum marker of bone collagen degradation. *Clin. Chem.*, **39**, 635–640.

STEWARD AF AND BROADUS AE. (1990). Clinical review 16: parathyroid hormone-related proteins: coming of age in the 1990s. *J Clin. Endocrinol. Metab.*, **71**, 1410–1414.

STOLL BA. (1983). Natural history, prognosis and staging of bone metastases. In *Bone Metastases Monitoring and Treatment*, Stoll BA, Parbho S. (eds), pp. 1–20. Raven Press: New York.

TERZOLO M, PIOVESAN A, OSELLA G, PIA A, REIMONDO G, POZZI C, RAUCCI C, TORTA M, PACCOTTI P AND ANGELI A. (1993). Serum levels of bone GLA protein (osteocalcin, BGP) and carboxyterminal propeptide of type I procollagen (PICP) in acromegaly: effects of long term octreotide treatment. *Calcif. Tissue Int.*, **52**, 188–191.

UEBELHART D, GINEYTS E, CHAPUY MC AND DELMAS PD. (1990). Urinary excretion of pyridinium crosslinks: a new marker of bone resorption in metabolic bone disease. *Bone & Mineral*, **8**, 87–96.

URWIN GH, PERCIVAL RC, HARRIS S, BENETON MNC, WILLIAMS JL AND KANIS JA. (1985). Generalised increase in bone resorption in carcinoma of the prostate. *Br. J. Urol.*, **57**, 721–723.